# Supplementary material for: Effects of Film Thickness on the Residual Stress of Vanadium Dioxide Thin Films Grown by Magnetron Sputtering
Source: Materials (Basel). 2023 Jul 19;16(14):5093. doi: 10.3390/ma16145093 (PMC10386697; doi:10.3390/ma16145093)
Supplement: Supplementary file 1 [file materials-16-05093-s001.zip › materials-2345772-supplementary.pdf]

Supplementary Materials

(a)

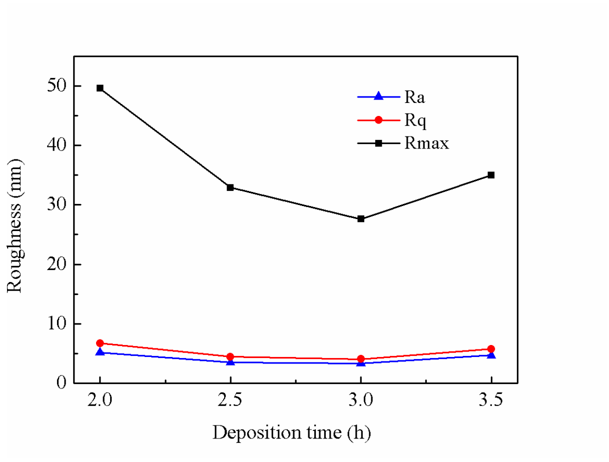

Figure S1. The surface roughness of VO_2 thin film measured by AFM

(b)

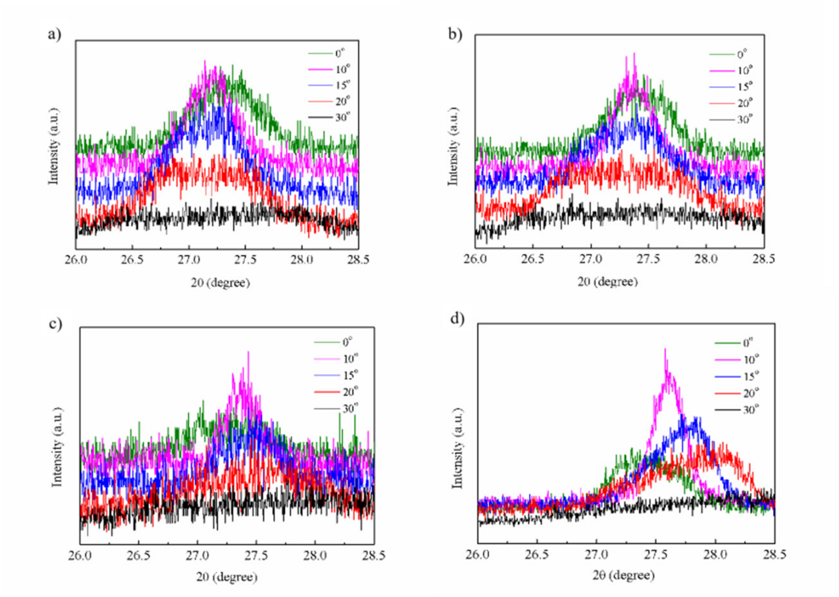

Figure S2. θ–2θ scans for reflection at different inclination angle ψ

(c)

The analytical model proposed by Tsui [50] was used to predict thermal stress can be written as:

$$\sigma_{the} = \frac{E_{ef}(\alpha_s - \alpha_f)\Delta T}{1 + 4(\frac{E_{ef}}{E_{es}})(\frac{h}{H})} \tag{1}$$

where are effective elastic

where $E_{ef} = E_f / (1 - \upsilon_f)$, $E_{es} = E_s / (1 - \upsilon_s)$, E_f, E_s, υ_f, υ_s, ΔT, h, H, α_f and α_s are effective elastic modulus of the thin film, effective elastic modulus of the substrate, elastic modulus of the thin film, elastic modulus of the substrate, Poisson's ratio of the thin film, Poisson's ratio of the substrate, difference in temperature, thin film thickness, substrate thickness, CTE of the substrate and thin film, respectively.

(d)

Numerical simulation of thermal stress on VO_2 thin film was calculated using ABAQUS. The thickness of thin film was fixed at 720nm. By substituting the temperature(0℃~300℃) in the FEM，the thermal stress was calculated as shown in Fig. S3. It can be seen that the thermal stress showed a phenomenon of "tensile-compressive stress conversion" with the temperature increase. The results reveal that the thermal stress is range from -0.21GPa to 0.023GPa. Therefore, the impact of thermal stress on the residual stress can be ignored.

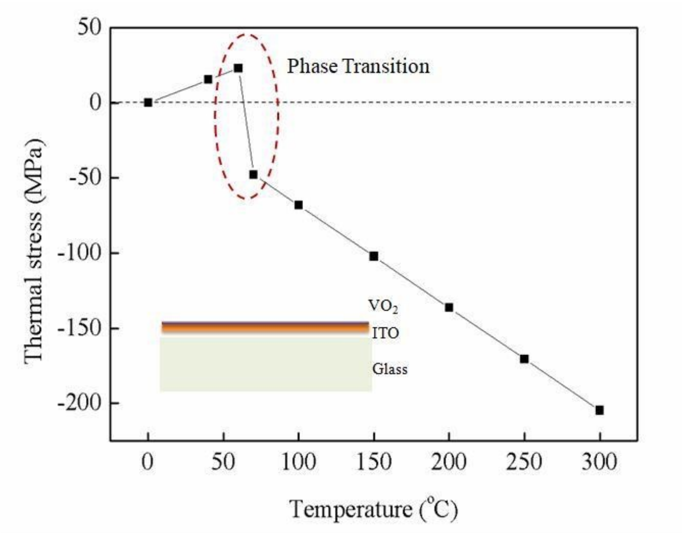

Figure S3. The thermal stress of VO2 thin film with 720 nm thickness
